# A systematic review of health sector responses to the coincidence of disasters and COVID-19

**DOI:** 10.1186/s12889-021-10806-9

**Published:** 2021-04-13

**Authors:** Sanaz Sohrabizadeh, Shiva Yousefian, Amirhosein Bahramzadeh, Mohammad Hossein Vaziri

**Affiliations:** 1 Safety Promotion and Injury Prevention Research Center, School of Public Health and Safety, Shahid Beheshti University of Medical Sciences, Tehran, Iran; 2 Department of Health in Disasters and Emergencies, School of Public Health and Safety, Shahid Beheshti University of Medical Sciences, Tehran, Iran; 3 Workplace Health Promotion Research Center, School of Public Health and Safety, Shahid Beheshti University of Medical Sciences, Tehran, Iran

**Keywords:** COVID-19 pandemic, Disasters, Health sector, Response

## Abstract

**Background:**

In December 2019, the Chinese city of Wuhan reported a novel pneumonia caused by COVID-19. While the COVID-19 pandemic has been increasingly affecting the world, the occurrence of disasters resulted in complex emergencies. The present review is aimed to identify the literature focused on health system response to coincidence of COVID-19 and disasters as well as describing their finding, implications and lessons-learned.

**Methods:**

This study was conducted and reported based on PRISMA guideline. The databases of Web of Sciences, PubMed, Scopus, Google Scholar and World Health Organization Library were searched. The inclusion criteria were all forms of published articles which investigated the coincidence of disasters and COVID-19 pandemic. Using the title and abstract screening, the selections of studies were performed by two researchers. Once, the relevant papers were finalized, the analysis was done in two parts of descriptive analysis and implications for health systems.

**Results:**

Out of 1245 studies generated by initial search, a number of 13 articles was selected for final analysis. Earthquake was the most frequent disaster which its coincidence with COVID-19 was studied by researchers (31%). The implications of researchers for healthcare system were explained in three sections of climatic events, earthquakes and all hazard approach in relation to COVID-19.

**Conclusion:**

Extracting the lessons learned from the regions affected by disasters at the time of COVID-19 pandemic can be helpful for healthcare professionals and policy-makers to improve their preparedness and response during disasters and a serious pandemic such as COVID-19. Further research is needed to identify the factors which strengthen the preparedness of health system for the dual risk of natural hazards and pandemics.

## Background

Disasters are catastrophic events with serious health, social, and economic consequences. Developing countries are disproportionately affected due to the insufficient resources, unsafe infrastructure, and lack of disaster preparedness systems [1]. Global population growth, poverty, and the urbanization in many countries have increased the number of people living in highly disaster-prone regions and multiplied their public health effects [2]. There is an imbalance between requests for healthcare services and the capacity of healthcare services to respond at the time of disasters. Furthermore, the response might be weakened due to destructed health care facilities, loss of medical equipment and logistics as well as healthcare personnel’s deaths and injuries [3].

In December 2019, the Chinese city of Wuhan reported a novel pneumonia caused by coronavirus disease (COVID-19), which has been spreading internationally. Coronavirus is one of the major pathogens that primarily target the human respiratory system. Previous outbreaks of coronaviruses included the severe acute respiratory syndrome (SARS) and the Middle East respiratory syndrome (MERS) which have been previously characterized as an agent of great public health threat [4, 5]. While the COVID-19 pandemic has been increasingly affecting the world, the occurrence of disasters such as earthquakes, floods and tornadoes in several countries resulted in complex emergencies. For instance, a number of 15 earthquakes in China at the time of COVID-19 epidemic aggravated the fears and anxiety of Chinese people. Flash floods, bushfires, and dust storms of Australia, killed at least 33 people and more than a billion animals, and destroyed thousands of homes in 2020 [6]. In America, since the start of the COVID-19 pandemic, several disasters have occurred including dozens of tornados in southern states; earthquake in Nevada; tropical storm Cristobal in Florida; flash-floods in Wisconsin; and Hurricane Hanna in Texas Gulf Coast [7]. Flash floods occurred in the southern provinces of Iran resulted in some deaths and considerable destructions on March 2020. The coincidence of COVID-19 and Tehran earthquake resulted in serious stress, anxiety and panic reactions which caused injuries and damages [8, 9].

Disasters can impose multiple pressures on the health systems and cause disruptions for providing health services [7, 10]. Furthermore, disasters-affected people live in temporary overcrowded shelters with inadequate health facilities and insufficient ventilation. Such conditions can exacerbate the COVID-19 pandemic due to the increase the increase of communicable diseases which are transmitted by water, air and vectors [11]. Accordingly, COVID-19 pandemic can significantly affect the disaster exposure and vulnerability as well as capacities of health systems [12, 13].

The literature reported that the risk of communicable diseases have increased during and after disasters. For instance, several researches declared that post-disaster changes such as stress, hygiene, and environmental issues have caused various infectious diseases in disaster-stricken regions [14–16]. Although the effects of disasters and COVID-19 pandemic on the people’s health highlight an urgent need for merging disaster risk reduction strategies into response plans [10, 17, 18], the health system preparedness and response in face to dual risk of disasters and COVID-19 have studied insufficiently. Therefore, the present systematic review is aimed to identify the literature focused on health system response to coincidence of COVID-19 and disasters as well as describing their finding, implications and lessons-learned.

## Methods

### Databases and search strategy

The databases of Web of Sciences, PubMed, Scopus, Google Scholar and World Health Organization Library were searched using related keywords selected through PubMed MeSH terms, similar articles and documents as well as experts suggestions. Reference lists of relevant articles and systematic reviews were searched as well. The main search term included COVID-19, disasters, health, health system, preparedness, and disaster management. The strategy of search was made by using AND/OR between selected terms and keywords (Table 1). In order to increasing the probability of identification of all relevant literature, the keywords were finalized based on the agreement among three researchers. The search terms were used in titles, abstracts, text, and keywords of articles. The literature search was conducted in September 2020.

**Table 1:** Example of selected databases and their applied search syntax

Database	Search Syntax	N
**PubMed**	(“Natural Disaster*” OR “Natural Hazard*” OR earthquak* OR Flood* OR Storm* OR Drought* OR Hurricane* OR Tsunam* OR Tornado* OR Cyclone* OR Cyclones* OR Wildfir*) AND (“COVID-19” “COVID 19” COVID19 OR Coronavirus* OR corona) NOT “cytokine storm*” AND (“Health system*” OR health sector)	296
**Scopus**	(ALL (“Natural Disaster” AND “Covid 19”) OR ALL (“Natural Hazard” AND “Covid 19”) OR ALL (“Natural Disasters” AND “Covid 19”) OR ALL (“Natural Hazards” AND “Covid 19”) OR ALL (earthquake AND “Covid 19”) OR ALL (earthquakes AND “Covid 19”) OR ALL (Flood AND “Covid 19”) OR ALL (Floods AND Covid19) OR ALL (Flash Flood AND Covid19) OR ALL (Flash Floods AND Covid19) OR ALL (Drought AND “Covid 19”) OR ALL (Storm AND “Covid 19”) OR ALL (Storms AND “Covid 19”) OR ALL (“Surge Storm” AND “Covid 19”) OR ALL (Surge Storms AND “Covid 19”) OR ALL (Landslide AND “Covid 19”) OR ALL (Landslides AND “Covid 19”) OR ALL (Fire AND “Covid 19”) OR ALL (Fires AND “Covid 19”) OR ALL (Wildfire AND “Covid 19”) OR ALL (Wildfires AND “Covid 19”) OR ALL (Hurricane AND “Covid 19”) OR ALL (Hurricanes AND “Covid 19”) OR ALL (Tornado AND “Covid 19”) OR ALL (Tornados AND “Covid 19”) OR ALL (Tsunami AND “Covid 19”) OR ALL (Tsunamis AND “Covid 19”) OR ALL (Cyclone AND “Covid 19”) OR ALL (Cyclones AND “Covid 19”) OR ALL (“Natural Disaster” AND “Covid 19 Coronavirus”) OR ALL (“Natural Hazard” AND “Covid 19 Coronavirus”) OR ALL (“Natural Disasters” AND “Covid 19 Coronavirus”) OR ALL (“Natural Hazards” AND “Covid 19 Coronavirus”) OR ALL (Earthquake AND “Covid 19 Coronavirus”) OR ALL (Earthquakes AND “Covid 19 Coronavirus”) OR ALL (Flood AND “Covid 19 Coronavirus”) OR ALL (Floods AND “Covid 19 Coronavirus”) OR ALL (Flash Flood AND “Covid 19 Coronavirus”) OR ALL (Flash Floods AND “Covid 19 Coronavirus”) OR ALL (Drought AND “Covid 19 Coronavirus”) OR ALL (Storm AND “Covid 19 Coronavirus”) OR ALL (Storms AND “Covid 19 Coronavirus”) OR ALL (“Surge Storm” AND “Covid 19 Coronavirus”) OR ALL (Surge Storms AND “Covid 19 Coronavirus”) OR ALL (Landslide AND “Covid 19 Coronavirus”) OR ALL (Landslides AND “Covid 19 Coronavirus”) OR ALL (Fire AND “Covid 19 Coronavirus”) OR ALL (Fires AND “Covid 19 Coronavirus”) OR ALL (Wildfire AND “Covid 19 Coronavirus”) OR ALL (Wildfires AND “Covid 19 Coronavirus”) OR ALL (Hurricane AND “Covid 19 Coronavirus”) OR ALL (Hurricanes AND “Covid 19 Coronavirus”) OR ALL (Tornado AND “Covid 19 Coronavirus”) OR ALL (Tornados AND “Covid 19 Coronavirus”) OR ALL (Tsunami AND “Covid 19 Coronavirus”) OR ALL (Tsunamis AND “Covid 19 Coronavirus”) OR ALL (Cyclone AND “Covid 19 Coronavirus”) OR ALL (Cyclones AND “Covid 19 Coronavirus”)) AND (ALL (Health) OR ALL (Healthcare) OR ALL (“Health system”) OR ALL (“Health sector”) OR ALL (“public health sector”) OR ALL (“health service”) OR ALL (“healthcare service”)) AND (ALL (Preparedness) OR ALL (“Disaster Preparedness”) OR ALL (Crisis Preparedness) OR ALL (“Crisis Management”) OR ALL (“Crises Management”) OR ALL (“Risk Management”) OR ALL (“Disaster Management”) OR ALL (“Risk Reduction”))	560
**Web of Sciences**	(TS = (“Natural Disaster” AND “Covid 19”) OR TS = (“Natural Hazard” AND “Covid 19”) OR TS = (“Natural Disasters” AND “Covid 19”) OR TS = (“Natural Hazards” AND “Covid 19”) OR TS = (earthquake AND “Covid 19”) OR TS = (earthquakes AND “Covid 19”) OR TS = (Flood AND “Covid 19”) OR TS = (Floods AND Covid19) OR TS = (Flash Flood AND Covid19) OR TS = (Flash Floods AND Covid19) OR TS = (Drought AND “Covid 19”) OR TS = (Storm AND “Covid 19”) OR TS = (Storms AND “Covid 19”) OR TS = (“Surge Storm” AND “Covid 19”) OR TS = (Surge Storms AND “Covid 19”) OR TS = (Landslide AND “Covid 19”) OR TS = (Landslides AND “Covid 19”) OR TS = (Fire AND “Covid 19”) OR TS = (Fires AND “Covid 19”) OR TS = (Wildfire AND “Covid 19”) OR TS = (Wildfires AND “Covid 19”) OR TS = (Hurricane AND “Covid 19”) OR TS = (Hurricanes AND “Covid 19”) OR TS = (Tornado AND “Covid 19”) OR TS = (Tornados AND “Covid 19”) OR TS = (Tsunami AND “Covid 19”) OR TS = (Tsunamis AND “Covid 19”) OR TS = (Cyclone AND “Covid 19”) OR TS = (Cyclones AND “Covid 19”) OR TS = (“Natural Disaster” AND “Covid 19 Coronavirus”) OR TS = (“Natural Hazard” AND “Covid 19 Coronavirus”) OR TS = (“Natural Disasters” AND “Covid 19 Coronavirus”) OR TS = (“Natural Hazards” AND “Covid 19 Coronavirus”) OR TS = (Earthquake AND “Covid 19 Coronavirus”) OR TS = (Earthquakes AND “Covid 19 Coronavirus”) OR TS = (Flood AND “Covid 19 Coronavirus”) OR TS = (Floods AND “Covid 19 Coronavirus”) OR TS = (Flash Flood AND “Covid 19 Coronavirus”) OR TS = (Flash Floods AND “Covid 19 Coronavirus”) OR TS = (Drought AND “Covid 19 Coronavirus”) OR TS = (Storm AND “Covid 19 Coronavirus”) OR TS = (Storms AND “Covid 19 Coronavirus”) OR TS = (“Surge Storm” AND “Covid 19 Coronavirus”) OR TS = (Surge Storms AND “Covid 19 Coronavirus”) OR TS = (Landslide AND “Covid 19 Coronavirus”) OR TS = (Landslides AND “Covid 19 Coronavirus”) OR TS = (Fire AND “Covid 19 Coronavirus”) OR TS = (Fires AND “Covid 19 Coronavirus”) OR TS = (Wildfire AND “Covid 19 Coronavirus”) OR TS = (Wildfires AND “Covid 19 Coronavirus”) OR TS = (Hurricane AND “Covid 19 Coronavirus”) OR TS = (Hurricanes AND “Covid 19 Coronavirus”) OR TS = (Tornado AND “Covid 19 Coronavirus”) OR TS = (Tornados AND “Covid 19 Coronavirus”) OR TS = (Tsunami AND “Covid 19 Coronavirus”) OR TS = (Tsunamis AND “Covid 19 Coronavirus”) OR TS = (Cyclone AND “Covid 19 Coronavirus”) OR TS = (Cyclones AND “Covid 19 Coronavirus”)) AND (TS = (Health) OR TS = (Healthcare) OR TS = (“Health system”) OR TS = (“Health sector”) OR TS = (“public health sector”) OR TS = (“health service”) OR TS = (“healthcare service”)) AND (TS = (Preparedness) OR TS = (“Disaster Preparedness”) OR TS = (Crisis Preparedness) OR TS = (“Crisis Management”) OR TS = (“Crises Management”) OR TS = (“Risk Management”) OR TS = (“Disaster Management”) OR TS = (“Risk Reduction”))	4

### Inclusion and exclusion criteria

The research team applied inclusion and exclusion criteria for selecting relevant studies. The inclusion criteria were determined as all forms of published articles (e.g. original paper, letter to editor, case study and commentary) which investigated the dual risk or coincidence of disasters and COVID-19 pandemic in the world. The exclusion criteria included documents were not published in English, and online news and reports as well as articles which their abstracts could not be accessed.

### Studies selection and analysis process

Using the title and abstract screening, and given the inclusion criteria, the selection of studies were performed by two researchers. In the next step, the full texts of the remaining articles were analyzed independently by two researchers. The discrepancies were discussed and in cases without any agreement, a third person was the final decision maker to include the documents. The studies that met one of the exclusion criteria were rejected. Included documents were checked by a checklist made by researchers and their qualities were recognized as suitable.

Once the studies selection was completed and the relevant papers was finalized, primary analysis was done to clarify the main characteristics of the selected literature. Accordingly, for all documents, authors were developed a checklist based on four main sections of setting, study design, findings and implication for health system. Then, the data extraction sheet was designed by each study’s information such as title, name(s) of the author(s), publication year, data source, journal’s name and article type. All extracted data was evaluated by members of the research team to verify accuracy and completeness. In order to manage the citations, EndNote software, version 17, was used, and all duplicated records were excluded. The present systematic review was conducted and reported based on PRISMA checklist.

## Results

### Description of included studies

**Fig. 1:**
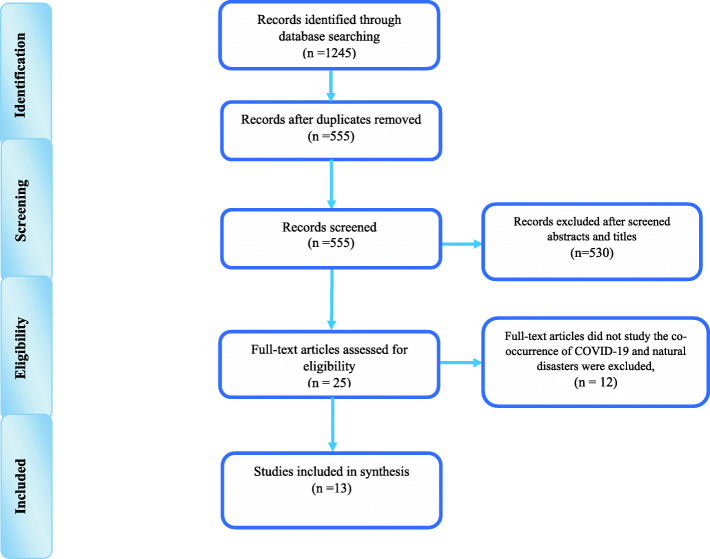
Article screening flowchart based on the PRISMA guideline to select final articles for analysis

A number of 1245 studies was generated by initial search. After removing the duplication, 555 references were identified for screening. Out of 555 extracted studies, 530 articles were removed due to meeting the exclusion criteria. The full texts of 25 studies were read and 13 articles which included the co-occurrence of disasters and COVID-19 as well as implications for health system were selected for final analysis (Fig. 1) (Table 2).

**Table 2:** The characteristics of the final selected articles

No	Title	Article type	Disaster type/time (2020)	Country (region)	Health effects
1	The COVID-19 Pandemic and Wildfire Smoke: Potentially Concomitant Disasters [19]	Editorial	Wildfire/wildfire season	Australia Western North America	Physical health; Environmental health
2	Stay home while going out – Possible impacts of earthquake co-occurring with COVID-19 pandemic on mental health and vice versa [20]	Editorial	Earthquake/ March	Republic of Croatia	Mental health
3	Mitigating the Twin Threats of Climate-Driven Atlantic Hurricanes and COVID-19 Transmission [21]	Policy analysis	Hurricane/ Atlantic hurricane season	United States and Canada	Healthcare system
4	Living in a Multi-Risk Chaotic Condition: Pandemic, Natural Hazards and Complex Emergencies [7]	Original research	floods/ June and July/	China	Healthcare system
			Earthquake/ March	Croatia	
			Earthquake/ May	Iran	
			Tropical Storm/ May	Amanda	
			Cyclone Harold/ April	Islands, Vanuatu, Fiji, and Tonga	
			Wildfire / May and June	Iran	
5	From natural disaster to pandemic: A health system pharmacy rises to the challenge [22]	Case report	Tornado/ March	United States	Health system pharmacy
6	Floods in China, COVID-19, and climate change [23]	Editorial	Floods/ June and July	China	Health system
7	Earthquake in the time of COVID-19: The story from Croatia (CroVID-20) [24]	View point	Earthquake/ March	Croatia	Healthcare system
8	COVID-19 pandemic and Zagreb earthquakes as stressors in patients with temporomandibular disorders [25]	Original research	Earthquake/ March	Croatia	Physical health
9	COVID-19 jeopardizes the response to coming natural disasters [10]	Editorial	All hazards	The world	Health system
10	Cascading Risks of COVID-19 Resurgence During an Active 2020 Atlantic Hurricane Season [26]	View point	Hurricane/ Summer	USA coastal states, PuertoRico and the USV irgin Islands	Healthcare system
11	An Eye on Covid: Hurricane Preparedness at a COVID-19 Alternative Care Site [27]	Case report	Hurricane/ June	United States	Health system and facilities
12	A multi-hazards earth science perspective on the COVID-19 pandemic: the potential for concurrent and cascading crises [28]	Original research	Earthquake/ March	Croatia	Health system
			Cyclone Harold/ April	Islands, Vanuatu, Fiji, and Tonga	
			Tornadoes/ April	United States	
			Bushfire/ Summer	Australia’s	
13	The Southwest Monsoon During COVID-19 Pandemic: A Potential Concern [11]	Editorial	Flood/ Monsoon season	South Asian countries	Health system

The subjects of all 13 articles included the coincidence of COVID-19 and disaster as well as communities’ healthcare systems at the same time. Earthquake, flood, hurricane, tornado and wildfire were the disasters which their coincidence with COVID-19 had been investigated. Of these, earthquake was the most frequent disaster which its coincidence with COVID-19 was studied by researchers (31%). All selected articles were published between April and September 2020. Editorial articles had the highest rate of document type (38.5%) and policy analysis was published at least (7.7%). About 10 out of 13 articles (77%) were investigated the effects of COVID-19 and disasters coincidence on healthcare system and had implications for the health system rather than mental or physical health (Table 3).

**Table 3:** The frequency of hazard type, article type and health effect

No	Subjects		N	(%)
1	**Hazard type**	Earthquake	4	31
		Flood	2	15.4
		Hurricane	3	23
		Tornado	1	7.6
		Wildfire	1	7.6
		All hazard approach	2	15.4
2	**Article type**	Editorial	5	38.5
		Original research	3	23
		Viewpoint	2	15.4
		Case report	2	15.4
		Policy analysis	1	7.7
3	**Health section**	Health system	10	77
		Physical/environmental health	2	15.4
		Mental health	1	7.6

### Health system response and preparedness measures

The included papers which have studied the co-occurrence of COVID-19 and disasters were considered in three sections of climatic events, earthquakes and all hazard approach.

#### Climatic events and COVID-19

A number of papers reported the co-occurrence of climatic events such as flood, hurricane and tornado and COVID-19 as the follow [11, 19, 21–23, 26, 27]:

The study of southwest monsoon in south Asian countries during COVID-19 pandemic found that a timely preparedness for monsoon season during ongoing COVID-19 is necessary. The authors stated that health system preparedness planning should be done by the participations of health staff and departments of disaster management in South Asian countries [11]. Similarly, the paper which reported the Hurricane occurred in USA during COVID-19 mentioned that the Louisiana Department of Health designed and activated the medical monitoring system (MMS) to lessen the pressure on hospitals overwhelmed by COVID-19 epidemic. Utilizing the patient safety and medical operations continuity as well as meeting the health needs of people affected by the Tropical Strom were the benefits of creating MMS [27]. Another study which studied the dual risk of hurricane and COVID-19 in the coastal regions of USA mentioned three steps for reducing the health consequences of COVID-19 and hurricane coincidence in the near future. The first step was to establish COVID-19 prevention lifestyle at the time of occurring convergent disasters. The second step consisted of strengthening communication in order to forming safer sheltering and evacuation in the regions affected by co-occurrence of COVID-19 and hurricane. The third step included learning from the lessons obtained from the coincidence of COVID-19 and the last storm (2020) to improve the disaster health management actions [26]. Similarly, the study which investigated the flood impacts during COVID-19 epidemic in China suggested urgent measures to prevent health and livelihood consequences of floods at the time of COVID-19 pandemic [23]. The study of COVID-19 and tornado coincidence in USA highlighted the pharmacy system response and preparedness including the external and multidisciplinary collaborations, effective communication among departments and teamwork. The authors stated that a strong disaster response system as well as effective collaborations and communications improved the functions of pharmacy system during COVID-19 epidemic and tornado [22].

The study of COVID-19 and hurricane in USA and Canada found that the anti-contagion policies and strategies as well as hygiene interventions need to be considered by public health managers and healthcare providers. Furthermore, releasing guidance and protocols on sheltering during COVID-19 pandemic, a 14-days self-quarantine of people who used mass shelters at the time of hurricanes, control of infections in shelter settings, checking symptom frequently, prepackaged meal and disinfection of surfaces as well as staying in assigned places at the time of sheltering were found as the health system preparedness and response measures [21]. The study of COVID-19 and wildfire in Australia and Western North America mentioned the necessity of healthcare providers’ preparedness for protecting patients and vulnerable people during wildfire season. The authors implied balancing the actions for responding to the dual risks of COVID-19 and wildfire events. Healthcare facilities need to be supplied and assessed by ventilation, heating and air conditioning experts to be prepared for the COVID-19 pandemic in the summer months [19].

#### Earthquakes and COVID-19

Several papers reported the health system response to co-occurrence of earthquakes and COVID-19 as the follow [20, 24, 25, 28]:

The authors of the co-occurrence of COVID-19 and earthquake study stated that the evacuation centers need to be available with the social distancing capacity as well as the availability of personal protective equipment and medical supplies such as respirators in regions affected by COVID-19. Furthermore, strengthening disaster coordination to inform COVID-19 epidemic response; identifying economic implication for COVID-19; improving community-based response and preparedness for coincidence of COVID-19 and disasters as well as preparing recovery plan at early phase were found as the health system preparedness and response measures [28]. The study of Croatia earthquake found that the risk of increasing the cases of COVID-19 in combination with an earthquakes is higher. Accordingly, the authors emphasized that a well-established health system as well as coordinated collaborations during outbreak response improve healthcare system preparedness and response [24].

The study of patients with temporomandibular disorders (TMD) in Croatia reported that COVID-19 and earthquakes played as stressors for TMD patients. TMD is defined as a “collective term that embraces a number of clinical problems that involve the masticatory muscles, temporomandibular joint, and the associated structures.” [29]. The authors mentioned the importance of the evaluation of psychological status of TMD patients who lived in earthquake affected regions [25]. Other study highlighted the effects of COVID-19 pandemic and disasters on people’s mental health in Croatia. The authors mentioned that pre and post-disaster context as well as people’s characteristics influenced the mental health interventions. It was found that resource allocation for mental health system activation in the regions affected by disasters and COVID-19 is important measure [20].

#### All hazard approach and COVID-19

Two included papers mentioned health system response to COVID-19 using all hazard approach as the follow [7, 10]:

The first paper found that the contingency planning and pre-crisis planning can reduce the health impacts of all hazards at the time of COVID-19 pandemic. Accordingly, contingency planning includes protocols for safe work, social distancing and analysis of worst-case scenarios based on the risk assessment. Furthermore, pre-disaster planning consists of coordination of stakeholders as well as optimizing resources for responding to potential effects of COVID-19 and upcoming disasters [10]. The authors of second paper found that any response to the cascading effects of disasters and COVID-19 pandemic needs to be considered in risk legislation and planning during disaster prevention and preparedness phases [7].

## Discussion

To the best of our knowledge, any systematic review has not been conducted about COVID-19 and disaster coincidence. The most reviews were studied COVID-19 models for hospital surge capacity planning [30], international public health responses to COVID-19 [31], police responses to COVID-19 [32] and mental health disorders at the time of COVID-19 outbreak [33]. Identifying the literature studied the health system response to coincidence of COVID-19 and disasters as well as describing their finding and implications and lessons-learned have been the aims of the current review. Based on the findings, earthquake was the frequent disaster occurred during COVID-19 pandemic and most of the literature focused health system response rather than individuals’ mental or physical health. The included studies were considered health system response to coincidence of COVID-19 and climatic events, earthquakes and all hazard approach. Accordingly, the discussion are described in three sections as the follow:

### Health system, COVID-19 and climatic disasters

A considerable number of included articles described health system responding to climatic disasters and COVID-19 coincidence. Based on the findings, preparedness and response planning, coordination, communication as well as establishing evaluation and monitoring structures (e.g. MMS) were mentioned as the main health system measures for managing the coincidence of COVID-19 and climatic disasters such as tornado, hurricane and flood. The health system needs to balance the reduction of COVID-19 transmission possibility and minimize the risk of climatic disasters such as hurricane exposure. In addition, safe evacuation and sheltering were suggested as the important measures during COVID-19 and disasters coincidence. Accordingly, World Health Organization (WHO) released implications for disaster evacuation shelters in the context of COVID-19. For instance, facilitating physical distancing such as putting up physical barriers, identifying and isolating suspected or confirmed cases of COVID-19, facilitating contact tracing within evacuation shelter, practicing proper hand washing, applying personal protective equipment, performing environmental cleaning as well as temperature and ventilation control were key public health principles suggested by WHO [34]. However, all important public health principles for preventing COVID-19 outbreak need to be educated by health system in disaster affected communities. Considering and following the public health surveillance steps such as case identification, isolation, quarantine, and contact tracing can be crucial as well. Furthermore, educating evacuation and sheltering behaviors to people during COVID-19 and applying lessons learnt from the previous climatic disasters can be important implications for health systems during COVID-19 and disasters coincidence [21].

A number of conflicts may exist for responding to COVID-19 and disasters coincidence. For example, although surgical mask and cloth can prevent COVID-19 transmission, they provided limited protection from wildfire smoke [19]. Accordingly, balancing the actions for responding to COVID-19 and wildfire needs to be considered by public health providers. In addition, the preparedness for wildfire smoke requires the collaborative actions of public and environmental health experts as well.

### Health system, COVID-19 and earthquakes

Based on the findings, balancing the risks of COVID-19 and earthquake response and recovery actions may be the most important implication for health systems response to COVID-19 pandemic and earthquake co-occurrence. Preparedness and recovery plan, community-based response and preparedness as well as multi-sector collaborations can improve the health system readiness for responding to COVID-19 outbreak in earthquake-affected regions.

Our findings revealed that providing healthcare services for patients with chronic diseases is one of the important tasks of health systems at the time of earthquakes and COVID-19 coincidences. For instance, providing mental health services for TMD patients who lived in the regions affected by earthquakes during COVID-19 was highlighted as the necessary measure. Monitoring and focusing on mental health status of patients with chronic diseases and pains were reported by several studies [35, 36]. In addition, chronic health conditions including cancer, hypertension, diabetes, cardiovascular diseases were associated with severity or mortality in COVID-19 patients. Thus, patients with chronic health conditions require continuous access to crucial medical and healthcare services during complex emergencies [37–40]. A prepared health system can prevent more deaths and disabilities among patients with chronic illnesses and ensure the continuity of care. Strengthening primary health care systems which are close to community and can provide comprehensive package of services helps the continuity of care. For instance, essential medical care can be home-delivered for patients with chronic illnesses by a robust primary health care system. In addition, preparedness and response planning, considering adequate surge capacity by collaboration of private sector, supplying health system with impeccable logistics, promoting chronic disease surveillance system and medical technology are suggested for ensuring the continuity of care for patients with chronic diseases affected by COVID-19 epidemic and disasters [41, 42]. In addition, health system can develop effective tools for management of chronic diseases including real-time clinical registries as well as applying virtual social networks for supporting healthy lifestyle changes during COVID-19 [43].

### Health system, COVID-19 and all hazard approach

Based on the findings, the response and preparedness plans of COVID-19 and disasters were classified into two parts of contingency planning; and pre-crisis planning and coordination. The increase of international cooperation as well as improving disaster health management systems can help countries, especially developing ones, encounter dual risk of COVID-19 pandemic and all hazards. Additional financial resource allocation for conducting research and investigations in regions affected by disaster and COVID-19 pandemic can help strengthen health system resilience [7, 10]. A resilient health system can detect, monitoring and responding to any health threat and weakness such as epidemics and disasters. The evaluation and monitoring conducted by health systems can help understand the pain and disability problems of patients who were affected by co-occurrence of COVID-19 and disasters. Furthermore, a prepared health system has access to data for making effective health decisions during complex emergencies such as pandemics and disasters coincidences. In addition, improving human resource capacity as well as promoting financial protection against health costs in the highly disaster prone regions during COVID-19 outbreak are suggested for achieving a prepared health system for all hazards [41, 44, 45].

## Conclusion

While there is a little known about the co-occurrence of COVID-19 and a disaster, our findings may help governments to establish risk reduction plans and strategies for this issue. In addition, extracting the lessons learned from the regions affected by disasters at the time of COVID-19 pandemic can be helpful for the preparedness of healthcare professionals and policy-makers and effective responding to disasters and a pandemic such as COVID-19. Further research is needed to identify the factors improving preparedness of health system for the dual risk of natural hazards and pandemics. Furthermore, conducting community-based research projects in the regions affected by disasters during COVID-19 can provide the healthcare administrators useful information for planning and decision-making. In addition, future studies are needed to develop measurement tools for assessing the level of health system preparedness for the dual risk of pandemic and disasters.

## Data Availability

The datasets used and/or analyzed during the current study are available from the corresponding author on reasonable request.

## Abbreviations

MMS: Medical Monitoring Station
TMD: Temporomandibular
WHO: World Health Organization
